# Automatic Identification and Intuitive Map Representation of the Epiretinal Membrane Presence in 3D OCT Volumes

**DOI:** 10.3390/s19235269

**Published:** 2019-11-29

**Authors:** Sergio Baamonde, Joaquim de Moura, Jorge Novo, Pablo Charlón, Marcos Ortega

**Affiliations:** 1 Centro de investigación CITIC, Universidade da Coruña, 15071 A Coruña, Spain; sergio.baamonde@udc.es (S.B.); jnovo@udc.es (J.N.); mortega@udc.es (M.O.); 2 VARPA, Instituto de Investigación Biomédica de A Coruña (INIBIC), Universidade da Coruña, 15006 A Coruña, Spain; 3 Instituto Oftalmológico Victoria de Rojas, 15009 A Coruña, Spain; pablo@pchoptometria.com; 4 Hospital HM Rosaleda, 15701 Santiago de Compostela, Spain

**Keywords:** computer-aided diagnosis, retinal imaging, optical coherence tomography, epiretinal membrane

## Abstract

Optical Coherence Tomography (OCT) is a medical image modality providing high-resolution cross-sectional visualizations of the retinal tissues without any invasive procedure, commonly used in the analysis of retinal diseases such as diabetic retinopathy or retinal detachment. Early identification of the epiretinal membrane (ERM) facilitates ERM surgical removal operations. Moreover, presence of the ERM is linked to other retinal pathologies, such as macular edemas, being among the main causes of vision loss. In this work, we propose an automatic method for the characterization and visualization of the ERM’s presence using 3D OCT volumes. A set of 452 features is refined using the Spatial Uniform ReliefF (SURF) selection strategy to identify the most relevant ones. Afterwards, a set of representative classifiers is trained, selecting the most proficient model, generating a 2D reconstruction of the ERM’s presence. Finally, a post-processing stage using a set of morphological operators is performed to improve the quality of the generated maps. To verify the proposed methodology, we used 20 3D OCT volumes, both with and without the ERM’s presence, totalling 2428 OCT images manually labeled by a specialist. The most optimal classifier in the training stage achieved a mean accuracy of 91.9%. Regarding the post-processing stage, mean specificity values of 91.9% and 99.0% were obtained from volumes with and without the ERM’s presence, respectively.

## 1. Introduction

Advances in medical imaging are contributing to the growth of knowledge about human pathological conditions, allowing specialists to better understand and comprehend previously unknown human body structures that could not be observed beforehand without the use of external auxiliary tools. More precisely, the introduction of multiple eye-imaging techniques, such as Optical Coherence Tomography (OCT), Fluorescein Angiography (FA), or Near-Infrared fundus Reflectance (NIR) allowed for ophthalmologists to precisely identify and study a wider range of pathologies related to the eye.

OCT is a non-invasive image modality that generates high-resolution, cross-sectional, and in vivo visualizations of the retinal layer tissues. These capabilities place OCT imaging among the most used medical image modalities in the analysis of the eye fundus, facilitating the study of relevant eye structures, such as the different retinal layers [1,2], the vasculature [3], or the choroid [4], as reference. Additionally, the OCT image modality is capable of providing highly precise measurements of relevant characteristics of the eye fundus, such as the macular thickness [5], the optic nerve head size [6], or the choroidal thickness [7]. Similarly, the NIR image modality is a non-invasive technique that achieves higher depth in the study of the eye fundus, resulting in an improved identification of the sub-retinal features. NIR images depict choroidal and retinal vessels of the eye fundus, providing a visual representation of the macular surface and related lesions, such as the central serous chorioretinopathy, the pigment epithelial detachment, or the epiretinal membrane [8]. Likewise, NIR images present a general overview of the eye fundus that complements the information that is typically obtained from the respective OCT volumes.

The Epiretinal Membrane (ERM) is defined as a reflective, fibrocellular tissue that appears over the inner retinal surface. The ERM is usually asymptomatic, without any apparent identified cause. However, if the ERM is left untreated, it can cause visual difficulties, such as blurred vision or metamorphopsia [9]. Its most commonly prescribed treatment implies ERM peeling by means of pars plana vitrectomy, resulting in significant improvement in vision for the majority of affected patients [10].

Usually, ERM etiology is idiopathic, without any other ocular pathologies. However, the ERM can be present as a secondary factor in eye diseases, such as vitreous hemorrhage, retinal angioma, or Diabetic Macular Edema (DME). Additionally, risk factors such as old age, previous ocular surgeries, or history of ERM in the other eye should be considered for ERM appearance.

DME is a retinal disease with a high prevalence of related ERM appearance [11]. Hyperglycemia causes weakening in the numerous blood vessels in the eye region, provoking Diabetic Retinopathy (DR). As a result, the blood vessels can leak fluid into the retinal area, inducing swelling and thickening of the retinal macular region (edema) [12]. Furthermore, DME is the principal cause of vision loss among patients diagnosed with diabetes mellitus [13,14,15]. In fact, the prevalence of diabetes mellitus is continuously increasing in the general population [16], representing a highly relevant contributing factor for multiple causes of death, such as cardiovascular or renal diseases [17]. Nowadays, the development of new methods and advances in the medical imaging field contribute to a better understanding and diagnosis of multiple eye pathologies. The high-resolution and cross-sectional capabilities of the OCT image modality are highly desired for computerized proposals, allowing the creation of automatic frameworks to identify pathologies such as DME [18] or DR [19], improving the diagnostic, prognosis, and monitoring processes.

The properties of OCT images have been used to identify and categorize the different types of DME. Otani et al. [20] defined three patterns in different instances of DME: retinal swelling, cystoid macular edema, and serous retinal detachment. The posterior work of Panozzo et al. [21] proposed a more extensive classification of DME by considering five relevant characteristics of the DME regions: retinal thickness (RT), extension, volume, morphology, and epiretinal traction. In particular, considering the epiretinal traction parameter, they proposed four different grades of severity based on the hyper-reflective properties of the ERM: absence of hyper-reflectivity, continuous line adhered to the retina, continuous line with multiple adhesion points and retinal distortion, and antero-posterior traction associated with mild cystoid macular edema. Similarly, Hasan et al. [22] proposed a system for the classification of macular edema by extracting a set of clinically significant features from different modalities of retinal imaging. Stevenson et al. [23] proposed an ERM classification scheme based on clinically relevant results from OCT images and the etiology of the ERM. Previous OCT-based ERM classification schemes described in the works of Hwang et al. [24] and Konidaris et al. [25] only considered morphological characteristics of the ERM. However, Stevenson et al. proposed a classification process based on the central foveal thickness measurement and the inner segment ellipsoid band integrity, while also considering whether the ERM appearance was idiopathic, primary, or secondary.

**Figure 1:**
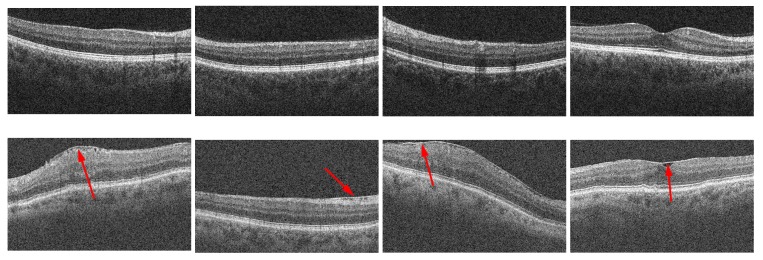
Multiple instances of 2D Optical Coherence Tomography (OCT) slices from different 3D OCT volumes with and without the presence of the Epiretinal Membrane (ERM). 1st row, OCT images without ERM’s presence. 2nd row, OCT images with ERM’s presence. The arrows point to the ERM’s presence, depicted as a hyper-reflective section on the Inner Limiting Membrane (ILM) layer.

As mentioned previously, the hyper-reflective characteristics of the ERM are typically used to discriminate between the ERM layer and the Inner Limiting Membrane (ILM) (Figure 1). The OCT image modality provides a highly accurate representation of the reflectivity values of the retinal region, facilitating the visual identification of the ERM. Traditionally, the classification of the ERM has been done following manual procedures, such as review processes by specialists or by the measurement of relevant studied features [26,27] in order to analyze and monitor the ERM’s presence after performing vitrectomy [28,29,30].

Wilkins et al. [31] automatically measured the central macular thickness by using manually defined boundaries around the studied region in individual 2D OCT slices. Similarly, other previous works on the matter [32,33] proposed an automatic methodology to identify the ERM’s presence in individual 2D OCT histological sections by extracting a heterogeneous set of features from the ILM layer region. To date, no proposed methodology entirely exploits the information of the 3D OCT volumes that may be obtained from the macular region of an individual patient. Previous proposals [23,24,25] focus on the definition of the ERM’s appearance and its characteristics, not performing a classification task that would serve to identify the ERM location over the retinal surface. Moreover, the mentioned proposals only exploit the information of individual 2D OCT slices without considering the entire 3D OCT volume and its useful spatial characteristics.

Given that, this work presents a completely automatic methodology for ERM identification and the generation of an intuitive map representation of the ERM’s presence using 3D OCT volumes over the macular region without any manual input. To do so, the proposal identifies the ILM layer and extracts a complete set of relevant domain-related, intensity, and texture-based features that have been proved to possess high discriminating value in ERM identification. Afterwards, we perform a feature selection step to reduce the dimensionality of the analyzed dataset. A set of instances representing the ERM’s presence and absence was used for the proposed classification task. Finally, we generate intuitive 2D reconstructions of the ERM layer for multiple 3D OCT volumes with ERM’s presence and absence. These results were finally refined and improved by exploiting information about the spatial relationship between neighboring 2D OCT slices. When available, the ERM identifications over the complete macular region are used together with the NIR image to offer a clear and intuitive visualization of the pathological ERM’s presence over the eye fundus to facilitate the work of the specialist. In summary, this fully automatic computational methodology represents an innovative proposal, including an extended definition and analysis of representative characteristics and classifiers combined with an accurate refinement of the identifications using spatial foveal information, as well as an intuitive output visualization for the posterior inspection of the specialists.

This work is divided into four different sections: Section 2 explains the various steps of the proposed methodology; Section 3 presents the practical results obtained, while also explaining the experimental tasks that were performed to validate the depicted outcomes; and Section 4 and Section 5 comment on the final results of the proposal and suggest multiple future lines of work on the matter.

## 2. Materials and Methods

**Figure 2:**
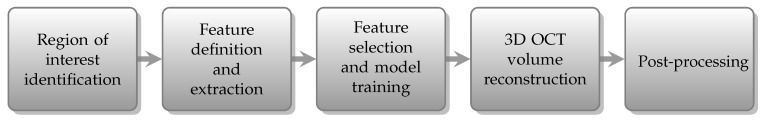
Main scheme of the proposed methodology.

The general scheme of the proposed methodology is illustrated in Figure 2. The inner limiting membrane (ILM) is defined as the region of interest (ROI) of the model, given the ERM is typically present over this retinal layer. Thus, in the first place, the ILM layer is segmented by means of an active contour model. Afterwards, we extracted a complete and heterogeneous set of features around the identifications of the ILM in the OCT images to characterize both the ERM’s presence and absence. A set of representative samples was used to train the proposed learning approach. In the next stage, we applied a feature selection method to identify the optimal subset of relevant features and optimize the process. After that, the optimal classifier was used to identify the presence of the ERM in 2D OCT slices. Putting together the output for each 2D OCT slice of the entire 3D OCT scan, we created a preliminary pathological map, that is, transforming the results into a 2D reconstruction of the ERM’s presence in the complete analyzed retinal area. Finally, a post-processing stage using a set of morphological operators was applied to improve the quality of the final extraction and reconstruction.

### 2.1. Acquisition

**Figure 3:**
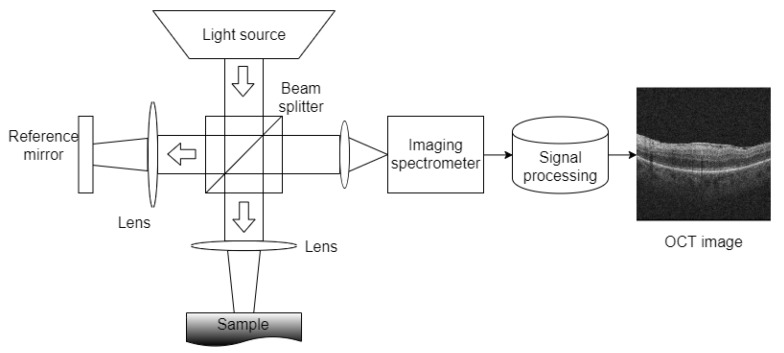
Schema of a basic Spectral Domain OCT (SD-OCT) system.

The Spectral Domain OCT (SD-OCT) imaging technique is based on low-coherence interferometry, more precisely on a Michelson interferometer. This configuration consists of two mirrors, one of them being, in this case, the tissue sample, and the other acting as a reference mirror. Reflected light from the tissue is combined with the reflected light from the mirror and passed through an imaging spectrometer in order to separate the spectral components at the output, as Figure 3 represents.

The areas of the analyzed sample that reflect higher amounts of light will create a higher amount of interference patterns than areas with lower reflective characteristics. This reflective profile, commonly known as an A-Scan, provides dimensional information about the analyzed sample. Similarly, a B-Scan, obtained by the combination of a series of A-Scans at different depths, provides cross-sectional information about the sample where the amplitudes of the reflective structures are normally represented in a gray-scale. Finally, a succession of B-Scans obtained rapidly and closely can be converted to a volumetric representation of the analyzed sample, known as a C-Scan representation.

### 2.2. Identification of the Region of Interest

**Figure 4:**
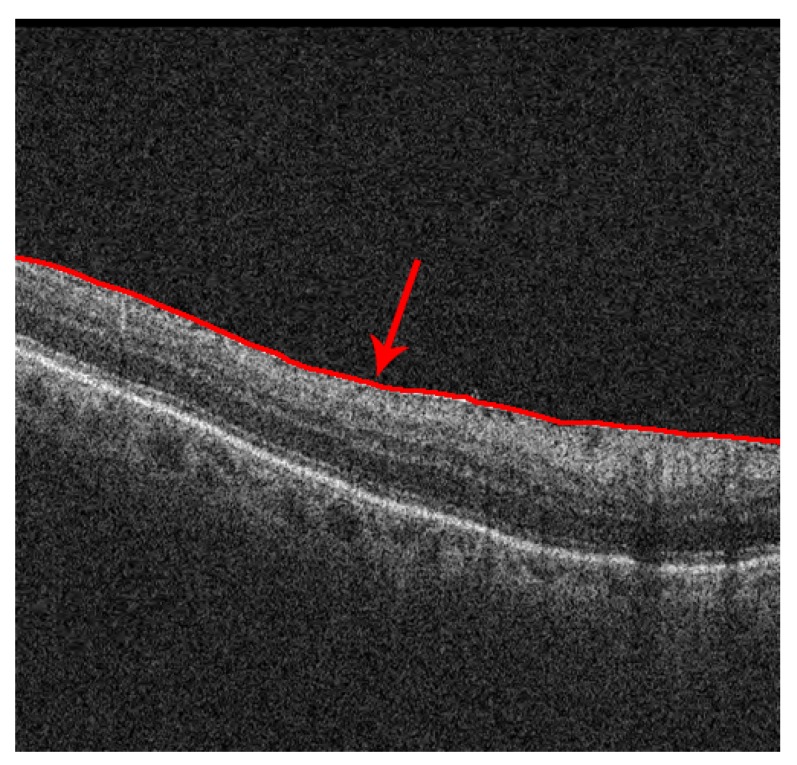
Localization of the region of interest. The arrow points to the segmented ILM layer.

The appearance of the ERM is restricted to the retinal surface, attached or detached from the ILM retinal layer. To define the ROI points, an active contour model (Snake) is used to obtain the precise shape of the ILM layer [34,35]. The Snake model acts by converging and adapting its shape around the points of minimal energy. The edges between the retinal structures and layers are zones of maximum gradient which minimize the energy value, and therefore a Snake model initialized at the top of the OCT image will converge to the superior layer of the retinal tissue, conforming to the ILM layer, as Figure 4 depicts.

### 2.3. Feature Definition and Extraction

**Figure 5:**
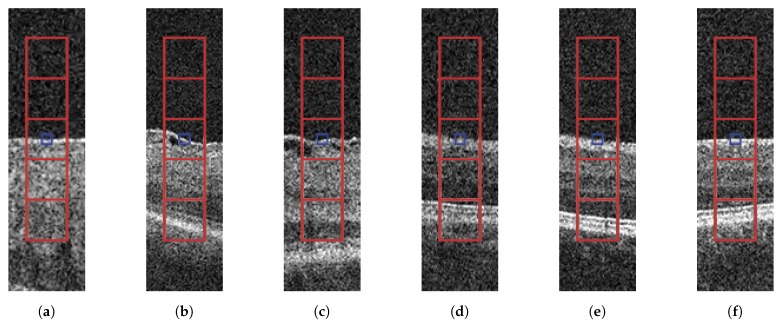
Regions of interest around the ILM layer points represented by the rectangular-shaped window and the five square-shaped windows. (**a**–**c**) Samples including ERM. (**d**–**f**) Samples without ERM.

The ERM can be identified by its reflective and structuring properties, appearing as a brighter region that is specifically attached or detached from the ILM layer. To validate this hypothesis, a vertical and rectangular-shaped window was defined around each ILM point to capture the differences across the different neighboring areas, such as the retinal background or the ILM layer. Each rectangular-shaped window was comprised of five square-shaped sub-windows that serve to perform a more precise analysis of the neighboring regions (Figure 5).

To validate this hypothesis, we used a complete and heterogeneous set of 452 intensity-, texture-, and domain-related features with high discriminative value that have been proved to maximize the amount of available information for each point of interest [33], briefly illustrated in Table 1:

### 2.4. Feature Selection and Model Training

The defined set of 452 features was large enough to contain redundant and irrelevant information on a subset of features. Also, the extraction of 452 features for each point of interest in all the 2D OCT slices of an entire 3D OCT scan significantly penalizes the efficiency of the system. To filter the irrelevant features from the complete set, we used a feature selection process to identify the ones that mostly contributed to the accuracy of the classification process.

The feature selection process was performed by the Spatial Uniform ReliefF (SURF) [36] algorithm, an extension of the ReliefF algorithm, which identifies the feature score differences between the *k* nearest neighbors and averages their contribution to the weights of each feature. In ReliefF, the optimal value of nearest neighbors to consider *k* has to be determined empirically, which is not feasible with the considerably large set of defined features and instances. In contrast, the SURF algorithm uses the average distance between instance pairs as a threshold to identify the nearest neighbors of each instance.

The reduced selected set of features was used to train multiple classifiers that have already been established as useful in many medical image analysis processes [37], such as the Random Forest classifier (RF), Support Vector Machines (SVM), and k-Nearest Neighbors classifier (kNN). The RF classifier assigns to each instance the most voted class from an aggregated set of decision trees. The SVM classifier constructs one or multiple hyperplanes in a multi-dimensional space to separate the data between the different labels, allowing non-linearly separable data to be differentiated. The kNN algorithm assigns the label of the majority of its *k*-nearest neighbors to each instance.

### 2.5. 3D OCT Volume Reconstruction and Map Generation

**Figure 6:**
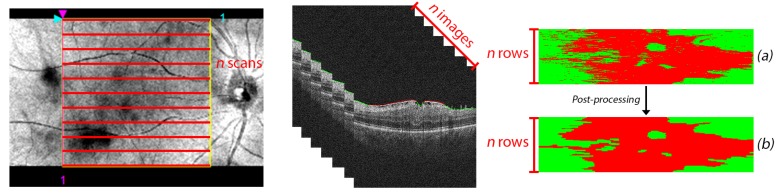
ERM classification process of a 3D OCT volume. A total of *n* OCT scans were generated for each patient, situated equidistantly in the complete retinal surface. The classification results for the *n* 2D OCT slices were arranged vertically in a reconstruction of height *n*, where each row represents the presence or absence of ERM in the associated 2D OCT slice. The post-processing stage improves the quality of the 2D map reconstruction by connecting neighboring pathological regions. (**a**) ERM classification before the post-processing map refinement stage. (**b**) ERM classification after the post-processing map refinement stage.

Later, the classification results obtained from the same 3D OCT volume were aggregated in a preliminary 2D reconstruction map of the ERM’s presence/absence in the retinal area. Each row of the resulting image describes the classification result of the respective OCT image in the full retinal volume, resulting in an accurate visual representation of the ERM extension over the macular region, as Figure 6 depicts.

### 2.6. Post-Processing Map Refinement

The ERM is mostly a uniform and continuous layer over the retinal surface, whereas the classification process labels each instance independently of the nearest neighbors (Figure 6a). To consider the spatial relation between the ERM points, we performed a post-processing stage where a set of adapted morphological operators were applied to the aforementioned reconstruction image, removing spurious detections and unifying nearby zones with ERM’s presence to provide coherence to the final ERM identification map, as well as to improve the results (Figure 6b).

In the initial step, we performed a connected-component analysis to identify the joint ERM zones, removing regions where their area was below a certain threshold, conserving only the larger connected ERM regions. Afterwards, we used a closing morphological operator to unify small background areas situated in those regions. This way, as said, we could produce more coherent and representative ERM regions in the output detection and representation of the proposed method.

## 3. Results

In this work, we used a total of 20 3D OCT volumes obtained from 20 different patients containing 2428 OCT images. From the total, 892 images were representative OCT cases including ERM’s presence, whereas 1536 OCT images portrayed cases without ERM’s presence. The 2D OCT slices were captured using a tomograph CIRRUS™ HD-OCT Zeiss using Spectral Domain Technology. The original image dimensions ranged from 490 × 490 to 510 × 510 pixels. Each of the 2428 2D OCT slices was labeled by an expert clinician, identifying the presence or absence of ERM and serving as ground truth for the entire training and validation process.

The training process was performed using a subset of five 3D OCT volumes for a total of 640 2D OCT slices, representing different sections of the retina. From this dataset, we randomly selected 16,206 training samples, while also preserving class balance between instances with ERM’s presence and absence. The remaining 15 3D OCT volumes were used to test the performance of the classification models.

To classify the input data, we used the RF, SVM, and kNN classifiers. For the kNN classifier, we generated three different configurations with *k* values of 2, 6, and 8, representing different numbers of nearest neighbors to be considered for the classification task.

Finally, the performance of the optimal classification model with respect to the classification of the 3D OCT volumes was improved by the post-processing stage. To this end, we obtained the optimal parameter configuration for the post-processing stage operations, calculating the overall performance of this stage for the complete set of 20 3D OCT volumes.

### 3.1. Feature Selection and Model Performance

As mentioned previously, we calculated the relevance of the defined features by using the SURF feature selector, ranking them from the most to the least relevant. Before identifying the optimal number of features to be used in the training task, we performed a hyper-parameter optimization task with a fixed number of the most relevant features to establish a baseline for each classification model.

**Figure 7:**
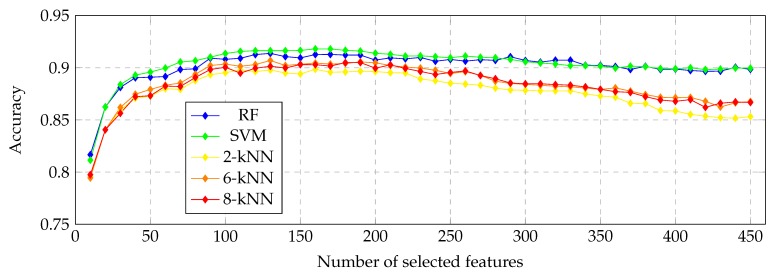
Evolution of the accuracy when increasing the number of used samples.

**Figure 8:**
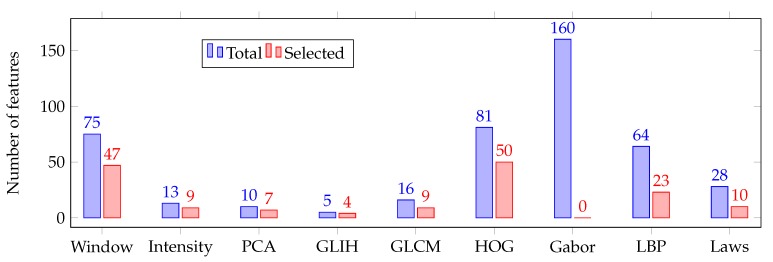
The 159 selected features for the most accurate classifier, grouped by categories.

These models were trained and tested using input sets with a variable number of relevant features. In order to avoid possible overfitting during the training process, we performed a 10-fold cross-validation, obtaining the mean accuracy of each resulting model. The evolution of the model accuracy is shown in Figure 7. Table 2 also describes the accuracy results in a limited region, also highlighting the most accurate performance for each model. Afterwards, we performed a fine-grain analysis to obtain the optimal number of features for each classifier type, as Table 3 depicts. As we can see, the stability of the feature dimensionality is clear, reaching numbers in all the cases between 140 and 200 features. In such cases, the stability is also significant in terms of the performance, reaching the best accuracy with the SVM but also with similar values for the rest of the analyzed classifiers. Figure 8 illustrates a breakdown of the 159 selected features with the most accurate model, emphasizing the selection of many domain-related and HOG features that capture the difference profiles and gradients of the ERM’s presence and absence, respectively.

### 3.2. Pathological Map Generation and Final Post-Processing Stage

The implemented ERM classification models are based on the individual classification of the ERM in isolated 2D OCT slices. However, the ERM has a consistent regional appearance as a uniform and compact area over the retinal surface. The lack of spatial information in the classification models generates lower and unrealistic output images, representing the ERM as individual points without any relationship between them, as previously represented in Figure 6a.

In order to offer a better and more realistic result, we proposed a post-processing stage including a series of consecutive morphological operations on the entire ERM identifications over all the 3D OCT volumes in order to exploit this spatial information of the consistent regional appearance of the ERM. The optimal configuration parameters for each operation were empirically obtained by exploring the complete parameter space for every operation, maximizing the relevant evaluation metric for the results. Additionally, there were no differences between optimizing the parameters globally or operation-wise. For this stage, we chose the Dice similarity coefficient [38] (Equation (3)) as the evaluation metric. The Dice similarity coefficient is widely used in image segmentation issues to compare output identifications [39], while also being most applied in binary classification problems.

**Figure 9:**
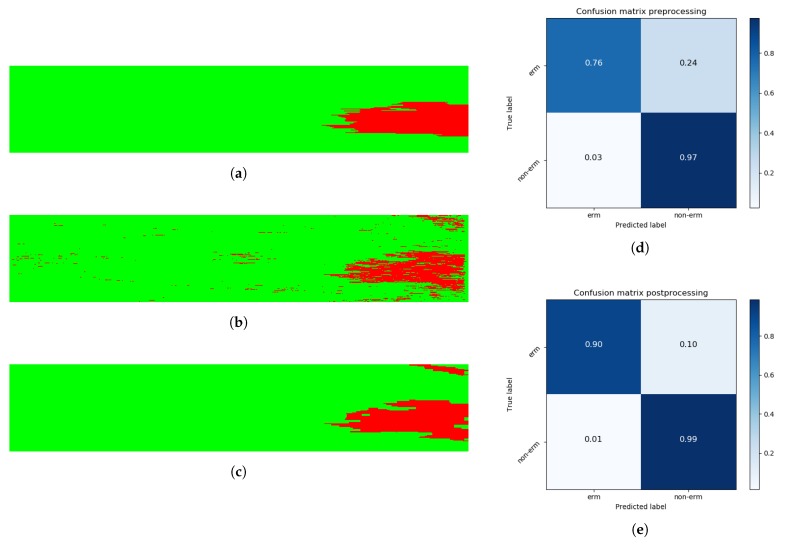
Complete result of a classification process. (**a**) The ground truth of the presence of ERM in the 3D OCT volume. Red pixels represent ERM instances, whereas green pixels symbolize non-ERM instances. (**b**) The resulting image from the automatic classification process. (**c**) The result after performing the post-processing stage. (**d**) The confusion matrix for the classification stage, resulting in Dice and Jaccard coefficients of 0.754 and 0.605. (**e**) Analogously, the confusion matrix after applying the defined post-processing operations, with increased Dice and Jaccard coefficients of 0.882 and 0.789, respectively.

**Figure 10:**
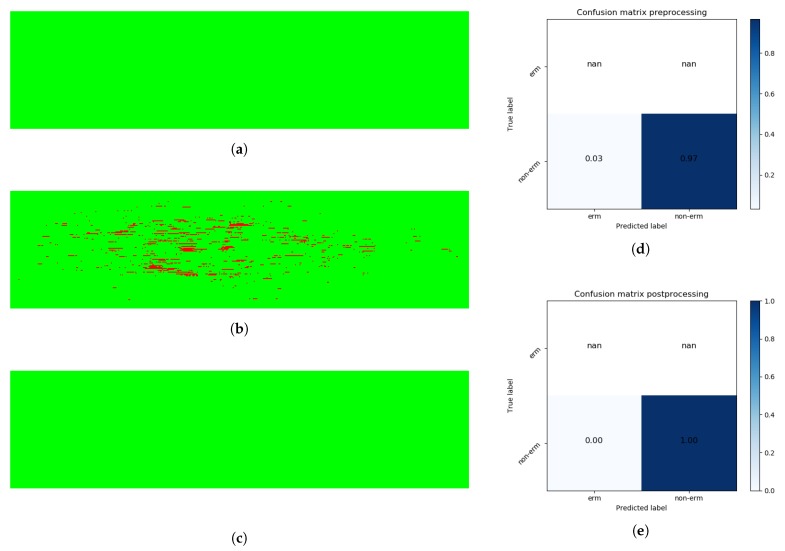
Classification of a 3D OCT volume from a healthy patient. (**a**) Illustration of how post-processing operations improve the quality of the final result by removing spurious false positive instances. (**b**,**c**) Representations of the confusion matrix for the classification and post-processing stages obtaining specificity values of 0.971 and 1.0. (**d**) The confusion matrix for the classification stage. (**e**) Analogously, the confusion matrix after applying the defined post-processing operations.

Table 4 shows a comparison of the classification results for each 3D OCT volume before and after the post-processing stage while also considering other relevant performance metrics, such as Sensibility (Equation (1)), Specificity (Equation (2)), and the Jaccard coefficient (Equation (4)). True Positive (TP) instances represent correct ERM classifications, whereas False Negative (FN) instances symbolize incorrect ERM classifications for areas without ERM’s presence in the ground truth. As a result, Sensitivity, the Dice similarity coefficient, and the Jaccard coefficient are not appropriate study metrics for 3D OCT volumes without ERM’s presence as their TP values are equal to zero. As we can see from the results, the contribution of the post-processing stage is significant, not only in terms of visual impact (Figure 6, Figure 9 and Figure 10), but also numerically. In particular, in the pathological OCT cases, we can see increments of the segmented ERM regions from 0.6695 and 0.5148 to 0.7799 and 0.6489 for the Dice and Jaccard coefficients, respectively.

(1) $$Sensitivity=\frac{TP}{TP+FN}$$

(2) $$Specificity=\frac{TN}{TN+FP}$$

(3) $$Dice=\frac{2TP}{2TP+FP+FN}$$

(4) $$Jaccard=\frac{TP}{TP+FP+FN}$$

## 4. Discussion

In comparison with the other ERM classification proposals, in this work we proposed a fully automatic computational proposal that exploits complete 3D OCT volumes to generate a fully automatic 2D map representation of the ERM’s surface. Moreover, the inclusion of spatial information in the post-processing stage improves the capacity of the method to precisely locate the ERM layer. Also, the suitability of the method was proved by the quality of the results that were obtained with three different representative classification schemes, demonstrating that the method is not reliant on a precise type of classifier.

Regarding the feature selection stage, the feature classes which were mostly selected are the domain-related window features and the HOG features. The domain-related features were specifically designed for this issue, correlating with the hypothesis that the bright intensities of the ERM’s presence can be detected by using information about the top and bottom neighboring areas around the ILM central point. Additionally, the characteristic gradient changes around the ERM’s presence when considering the luminosity of the layer in OCT images factors for the selection of the HOG features, due to their ability to identify gradient changes on the analyzed image. In contrast, the absence of relevant texture information around the retinal surface contributes to the lower significance of the texture-based descriptors, as happens with the Gabor filters.

Considering the classification experiments, despite the accurate performance of all the combinations, we concluded that the most accurate classifier was the SVM classifier with an accuracy of 91.9% when using the 159 most relevant features. We can point out that the SVM classifier obtains higher accuracy in the majority of the designed experiments, independently of the number of selected features by all the classifiers. In any case, as said, the highest accuracies are obtained when considering a number of features between 140 and 200, this number being significantly stable.

The classification model was applied to the complete dataset of 3D OCT volumes, obtaining a set of 2D images that represents the presence or absence of the ERM over the entire retinal surface. The resulting images provide highly accurate representations of the ERM extension across the retinal surface, as Figure 9 depicts. Even in 3D OCT volumes with more variability in their intensity values, the obtained results allow the specialist to quantify the overall presence of the ERM in the retinal surface. Additionally, applying the methodology to a 3D OCT volume from a patient without ERM’s presence (Figure 10) results in considerably higher-quality results, achieving specificity values higher than 0.98 for all the cases given the robustness of the method to recognize the ERM absence.

The results presented in Table 4 support the hypothesis of the suitability of the post-processing stage to improve the performance of the complete methodology, represented by the change of the mean Dice scores from 0.670 to 0.780 after the post-processing stage in 3D OCT volumes with ERM’s presence. Similar improvements are coherently present for the rest of the considered performance metrics and in the majority of the individual 3D OCT volumes. Additionally, the mean specificity of the classifier with the complete dataset of 3D OCT volumes (with and without the ERM’s presence) also improves in the post-processing stage, achieving a final specificity value of 0.955 at the post-processing stage, in contrast with the specificity value of 0.941 obtained in the previous classification stage. This demonstrates the imperfection of the individual classifications and significantly motivates and justifies the potential of improvement using the spatial information of the coherent regional ERM appearance.

**Figure 11:**
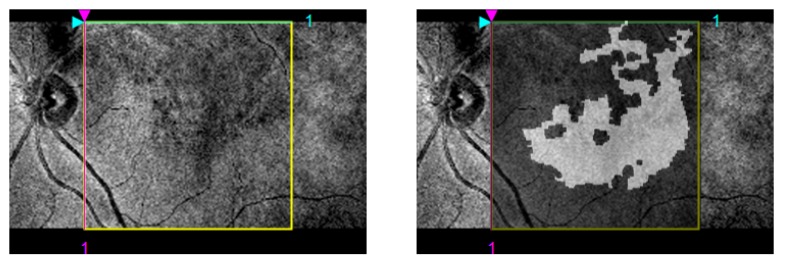
Visualization of the identified ERM regions combined with the NIR image for the same 3D OCT volume. The NIR image (left) represents the macular surface where the ERM is also located. The ERM reconstruction image fitted to the studied region in the NIR image. Darker areas in the NIR image are similarly situated in the ERM reconstruction and identified as areas with ERM’s presence (white).

In order to verify the diagnostic suitability of the 2D map reconstructions, we adapted the final 2D maps to the NIR images from each respective patient in the cases where this image modality was available. The NIR images represent the macular surface and contain exploitable information about the ERM’s presence. Figure 11 shows the similarity between the ERM surface portrayed by the 2D map representations and the NIR image, supporting the diagnostic suitability of the ERM map reconstruction.

In summary, the different experiments conducted demonstrate the suitability of the defined set of intensity, texture, and domain-related characteristics, which were refined by representative feature selectors and exploited by different classifiers. Also, the experiments corroborated the positive contribution of the post-processing refinement using the spatial foveal information. Complementarily, the final intuitive visualization for the posterior inspection of the specialists strengthen the novelty and potential of the proposed computational method.

## 5. Conclusions

The detection of the ERM is a highly relevant issue in the opthalmological field due to the association of the pathological ERM’s presence with other relevant eye diseases, such as diabetic macular edema or ocular inflammatory disease. Additionally, leaving the ERM untreated can cause a significant damage in the vision acuity, usually needing clinical intervention. For these reasons, early identification of the ERM can contribute to the reduction of future complications of pathology.

In this work, we presented a complete automatic methodology to identify the ERM’s presence in complete 3D OCT volumes. The method is fully automatic and does not require further human interaction at any stage, in contrast with previous methods in the field which require some interactions from a specialist at certain stages, such as the input of manual markers to locate the ROI. By using a complete set of heterogeneous domain-related, texture, and intensity features with high discrimination value, we obtained a significantly accurate performance of the implemented classification strategies. Furthermore, the introduction of spatial information from the complete 3D OCT volume demonstrated its suitability to increase the overall quality of the entire pathological identifications. The automatic methodology described using 3D OCT volumes has no other state-of-the-art references, which is why no further comparisons were made with other ERM identification proposals.

Two different validation stages were proposed for the complete methodology. Firstly, we selected 16,206 representative samples and 452 designed features that were obtained from a subset of five 3D OCT volumes in order to train and validate multiple representative classifiers with a variable number of relevant features, resulting in an accuracy score of 0.919 for the SVM classifier when using the 159 most relevant features. The post-processing task in the second validation stage refined the preliminary 2D map reconstructions obtained from the optimal classifier, achieving a final Dice score of 0.780 for the 3D OCT volumes with ERM’s presence and a final specificity score of 0.955 for the complete set of 3D OCT volumes (with and without ERM’s presence).

The obtained experimental results demonstrated the utility of our proposal in order to facilitate and reduce the workload of the clinicians in the identification of the ERM in 3D OCT volumes and analyze its characteristics. This proposal generates 2D image reconstructions of the retinal surface that integrate the ERM’s presence information from 3D OCT volumes, facilitating the diagnostic process. Additionally, the proposed system is highly tolerant to OCT image differences, obtaining adequate results in difficult analysis cases.

The suitability of the proposed approach for the automatic detection of the ERM serves as the initial basis for the complete identification and reconstruction of the ERM in 3D OCT volumes. In future works, we consider the further complete integration of the obtained results with NIR images to better validate the hypothesis of the proposed approach. Moreover, the ERM reconstruction output can be used to generate a 3D topographical map of the ERM’s presence in the complete retinal surface, further increasing the amount of available information at a glance. Additionally, the instances of ERM detached from the ILM can be studied separately, adding another layer of depth to the classification process and further increasing the discriminative power of the system. Finally, a higher amount of classification models can be integrated in the experimental process to account for further different approaches to the classification task.

## Figures and Tables

**Table 1:** Composition of the used feature set.

**Texture-Based Features**	Principal Component Analysis (PCA) features	10
	Gray-Level Co-occurrence Matrix (GLCM)	16
	Gabor features	160
	Local Binary Patterns	64
	Laws features	28
**Domain-Related Features**	Window features	75
**Intensity-Based Features**	Intensity global features	13
	Gray-Level Intensity Histogram (GLIH)	5
	Histogram of Oriented Gradients (HOG)	81

**Table 2:** Accuracy results with a different number of features. In bold, results of the most accurate configuration for each classifier.

Number of Features	20	40	60	80	100	120	140	160	180	200
RF	0.862	0.890	0.891	0.899	0.908	0.912	0.910	**0.913**	0.912	0.907
2-kNN	0.824	0.872	0.885	0.895	0.900	0.912	0.904	0.909	**0.911**	0.909
6-kNN	0.840	0.872	0.882	0.891	0.900	0.900	0.903	**0.905**	0.900	0.899
8-kNN	0.841	0.872	0.882	0.890	0.900	0.900	0.900	0.903	**0.905**	0.900
SVM	0.862	0.893	0.899	0.907	0.914	0.916	0.916	**0.918**	0.917	0.914

**Table 3:** Most accurate classifier of each type and corresponding optimal number of features. In bold, values indicating the most accurate configuration.

Classifier	RF	2-kNN	6-kNN	8-kNN	SVM
Number of features	137	182	183	184	159
Accuracy	0.914	0.911	0.906	0.906	**0.919**

**Table 4:** Mean Sensibility, Specificity, Dice and Jaccard coefficients for the complete dataset using the selected SVM classifier, divided by the experimental step.

		Classification Stage				Post-Processing Stage			
Patient Class	Identifier	Sensitivity	Specificity	Dice	Jaccard	Sensitivity	Specificity	Dice	Jaccard
ERM	**Mean**	0.7495	0.8944	0.6695	0.5148	0.8251	0.919	0.7799	0.6489
	**Std. Dev.**	± 0.1648	± 0.0652	± 0.1101	± 0.1403	± 0.1545	± 0.0544	± 0.0924	± 0.1277
Non-ERM	**Mean**	-	0.9880	-	-	-	0.9901	-	-
	**Std. Dev.**	-	± 0.0115	-	-	-	± 0.0061	-	-
